# Development and assessment of a novel magnetic nanoparticle antibody-conjugate and aptamer-based assay (MNp-Ab-Ap assay) for the rapid diagnosis of pleural tuberculosis

**DOI:** 10.7150/ntno.95332

**Published:** 2025-01-01

**Authors:** Pratibha Sharma, Rakesh Kumar Gupta, Simran Aittan, Divya Anthwal, Manisha Dass, Rakesh Yadav, Ashish Behera, Abhijeet Dhiman, Sahajal Dhooria, Sunil Sethi, Ritu Singhal, Puneet Arora, Ashutosh Nath Aggarwal, Tarun Kumar Sharma, Sagarika Haldar

**Affiliations:** 1 Translational Research Laboratory, Department of Experimental Medicine and Biotechnology, Post Graduate Institute of Medical Education and Research (PGIMER), Chandigarh, India.; 2 Department of Medical Microbiology, PGIMER, Chandigarh, India.; 3 Department of Internal Medicine, PGIMER, Chandigarh, India.; 4 Department of Biotechnology, All India Institute of Medical Sciences, New Delhi, India.; 5 Department of Pulmonary Medicine, PGIMER, Chandigarh, India.; 6 Department of Microbiology, National Institute of Tuberculosis and Respiratory Diseases, New Delhi, India.; 7 Department of TB and Respiratory Diseases, National Institute of Tuberculosis and Respiratory Diseases, New Delhi, India.; 8 Scientific Pursuit in Advancing Research and Knowledge (SPARK) Laboratory, Department of Medical Biotechnology, Gujarat Biotechnology University, Gujarat, India.

## Abstract

**Background:** Pleural tuberculosis (pTB) is a diagnostic challenge because of its non-specific clinical features, lack of accurate diagnostic tools and paucibacillary nature of the disease.

**Methods:** We, here describe the development of a novel magnetic nanoparticle antibody-conjugate and aptamer-based assay (MNp-Ab-Ap assay) targeting 4 different *Mycobacterium tuberculosis* (*M*.* tb.*) antigens (GlcB, MPT51, MPT64 and CFP-10) for pTB diagnosis. The MNp-Ab-Ap assay was developed by conjugating polyclonal antibodies on the surface of magnetic nanoparticles (MNPs) by using EDC-NHS chemistry. These conjugated MNPs were used to capture *M. tb.* antigens present in the pleural fluid samples. The resulting antigen-antibody complex was detected by antigen-specific 5'-biotinylated aptamers. All assays were standardized using samples of the 'Development set' (n=17) and evaluated in the 'Validation set' (n=114) in a blinded manner. Patient categorization was done using a 'Composite Reference Standard'. Assay cut-offs were determined from the 'Development set' (n=17; 'Definite & Probable' pTB; n=9 and 'Non-TB'; n=8) by calculating mean+3SD of OD_450_ values of the 'Non-TB' group and applied to 'Validation set' (n=114; 'Definite' pTB; n=8, 'Probable' pTB; n=34, 'Possible' pTB; n=28 and 'Non-TB'; n=44).

**Results:** Out of the 4 assays, MPT51-based MNp-Ab-Ap assay performed the best with 66.6% (95%CI;50.4-80.4) sensitivity and 95.4% (95%CI;85.1-99.4) specificity in the combined 'Definite and Probable' pTB group. Xpert MTB/RIF assay detected only six samples in the 'Validation set'. Binary logistic regression analysis indicated that MPT51-based MNp-Ab-Ap assay provided an incremental advantage over the existing diagnostic algorithm for pTB.

**Conclusions:** We conclude that MPT51-based MNp-Ab-Ap assay is a novel technique that can pave the way towards rapid and accurate diagnosis of pTB.

## Introduction

Pleural tuberculosis (pTB) is the second most frequent type of extrapulmonary tuberculosis (EPTB) in India 1. It is a diagnostic challenge because of its non-specific clinical features, paucibacillary nature of disease, and lack of sensitive diagnostic tools. Early and accurate diagnosis of pTB is important for its timely management to minimize morbidity and mortality 2, 3. In spite of the availability of several diagnostic tests, an accurate test for pTB is still elusive 4. The inadequacy of evidence-based microbiological techniques including the gold standard *Mycobacterium tuberculosis* (*M*. *tb.*) culture has led to the use of a composite reference standard (CRS) for pTB diagnosis. The CRS is a combination of clinical criteria along with results of radiological, cyto-biochemical, histological, microbiological and molecular assays 4. The WHO-endorsed Xpert MTB/RIF assay (Xpert) and its advanced version, Xpert Ultra also have an inadequate sensitivity (pooled sensitivity of 21% and 47% respectively against CRS, and 52% and 68% correspondingly against a microbiological reference standard) with specificity ranging from 97%-100% for pTB diagnosis 5. Therefore, in view of the non-availability of an accurate test for pTB diagnosis, there is a vital need for novel diagnostic tools. A biomarker-based rapid test is an urgent need for the diagnosis of both pulmonary TB and EPTB 6-8. We have recently described the diagnostic utility of cell-free *M*.* tb*. DNA as a pathogen-specific biomarker for pTB 9. The detection of *M*.* tb*.-specific antigens in the clinical sample could also provide a direct evidence of TB disease 10.

Aptamers have recently emerged as a promising tool in the field of TB diagnostics 11-13. They are synthetic oligonucleotides which act as chemical rivals of antibodies since they bind to their targets with high specificity and affinity due to their stable 3D-folded structures 14-16. As diagnostic reagents, they have been used in several platforms such as aptamer-linked immobilized sorbent assay (ALISA), electrochemiluminescence assay, dot-blot and nanoparticle based assays etc. for the detection of analytes 16. Here, we describe the development and evaluation of a magnetic nanoparticle antibody-conjugate and aptamer-based assay (MNp-Ab-Ap assay) for the detection of 4 different *M*.* tb*. specific antigens (GlcB, MPT51, MPT64 and CFP-10) in pleural fluid (PF) samples. These antigens were selected because of their immunodiagnostic potential in literature and their excellent performance for other forms of EPTB such as tuberculous meningitis 17, 18. In this assay, antibody-conjugated magnetic nanoparticles (MNPs) were used to efficiently capture the antigens in pleural fluid samples followed by the detection of resulting antigen-antibody complexes by antigen-specific DNA aptamers. The developed MNp-Ab-Ap assay was also compared with conventional indirect ELISA.

## Results

**Figure 1:**
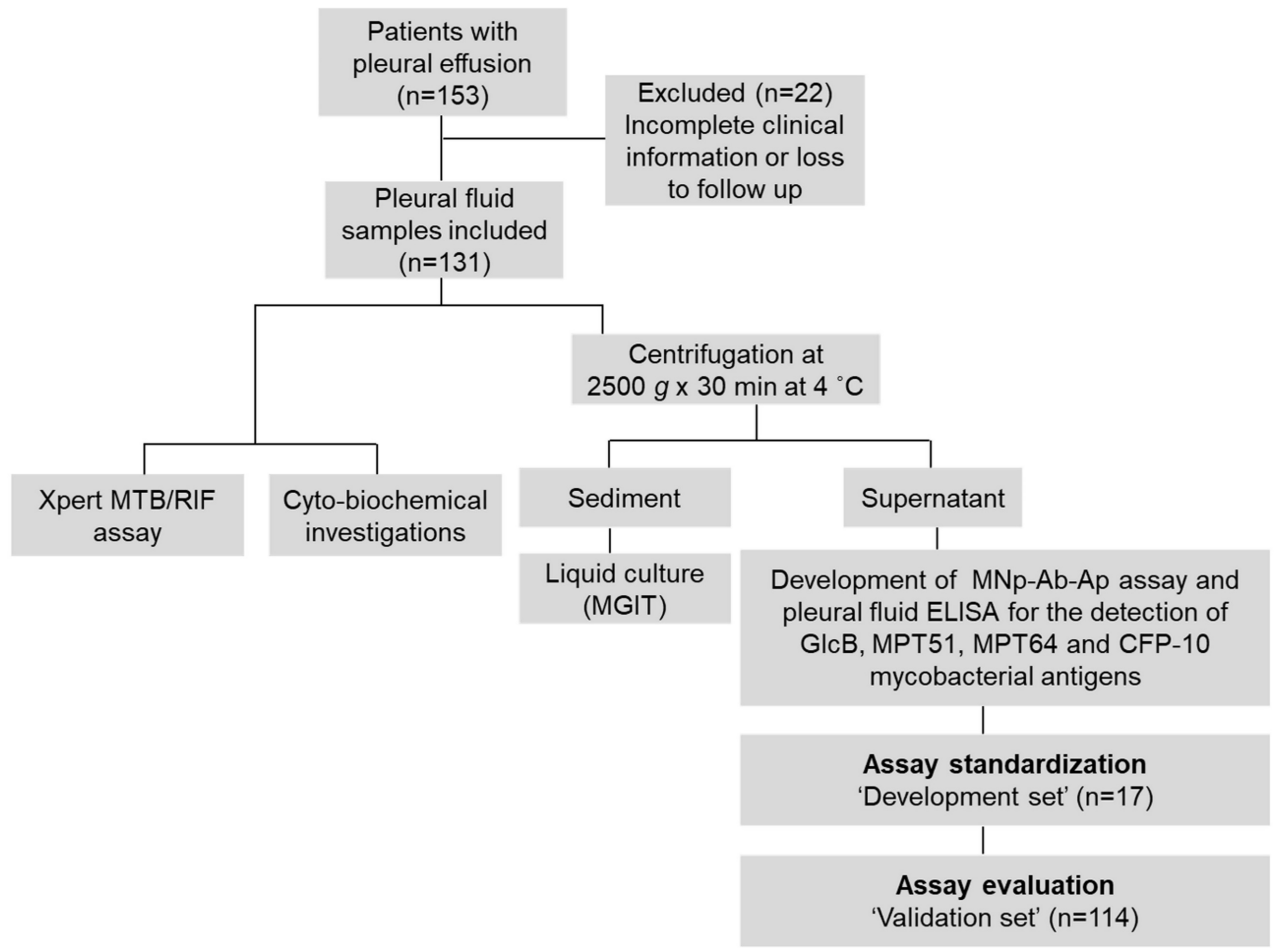
Flowchart of the study. MNpAb-Ap assay: Magnetic nanoparticle antibody-conjugate and aptamer-based assay.

**Clinical and laboratory findings.** Out of n=153 initially enrolled study participants, n=131 patients ['Development set' (n=17), and 'Validation set' (n=114)] were finally included in the study (Figure 1). In the 'Validation group', clinical symptoms such as fever, weight loss, chest pain, loss of appetite, cough, and fluid analysis i.e. lymphocytic predominance (> 50%), reduced glucose (< 60 mg/dL), high protein (> 2.5 g/dL) and elevated adenosine deaminase (ADA) values (> 33 U/L) were significantly associated with pTB disease (p < 0.05) (Supplementary Tables S1 and S2). The difference in the haemoglobin values, neutrophils, leukocytes, urea and creatinine were also found to be significant between the 'pTB' and 'Non-TB' group (Supplementary Table S2).

**Figure 2:**
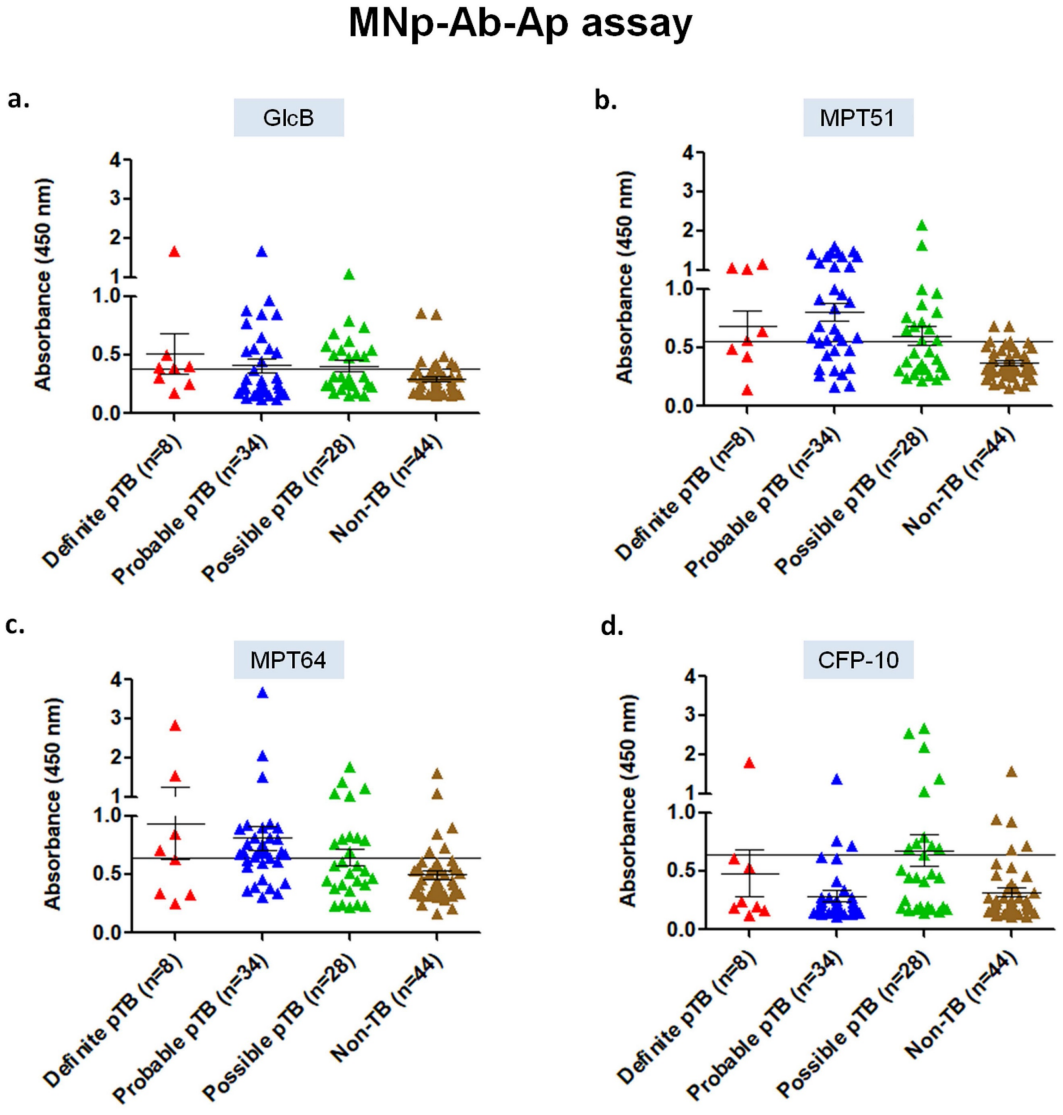
Scatterplots for OD_450_ values of MNp-Ab-Ap assay in pleural fluid samples of 'Validation set' (n=114) of the study. a) GlcB, b) MPT51, c) MPT64, and d) CFP-10. Horizontal line across the graph represents the cut-off generated from mean+3SD of the OD_450_ values of Non-TB samples of the 'Development set' (n=17) of the study.

**Assessment of MNp-Ab-Ap assay in pleural fluid samples for the 4 mycobacterial antigens.** The developed 5' biotinylated aptamers were first evaluated for specific binding with the purified protein antigens (GlcB, MPT51, MPT64 and CFP-10) captured by the MNp-Ab conjugates (Supplementary Figure S1). Once this was successfully assessed, the MNp-Ab-Ap assay was standardized on the 'Development set' (n=17), and subsequently applied on the 'Validation set' (n=114). In the 'Definite' pTB group, MPT51-based MNp-Ab-Ap assay performed the best with a sensitivity and specificity of 62.5% (95%CI;24.4-91.4) and 95.4% (95% CI;85.1-99.4) followed by MPT64 [sensitivity, 50% (95%CI;15.7-84.3); specificity, 86.3% (95%CI; 72.6-94.8)], GlcB [sensitivity, 62.5% (95%CI;24.4-91.4); specificity, 81.8% (95%CI;67.2-91.8)] and CFP-10 [sensitivity, 25% (95%CI;3.1-65); specificity, 88.6% (95%CI;75.4-96.2)]. Similar results were obtained in the combined 'Definite and Probable' pTB and 'Total' pTB group wherein MPT51 was the best antigen with a sensitivity of 66.6% (95%CI;50.4-80.4) and 58.5% (95%CI;46.1-70.2) respectively with similar specificity (Table 1, Figure 2).

**Figure 3:**
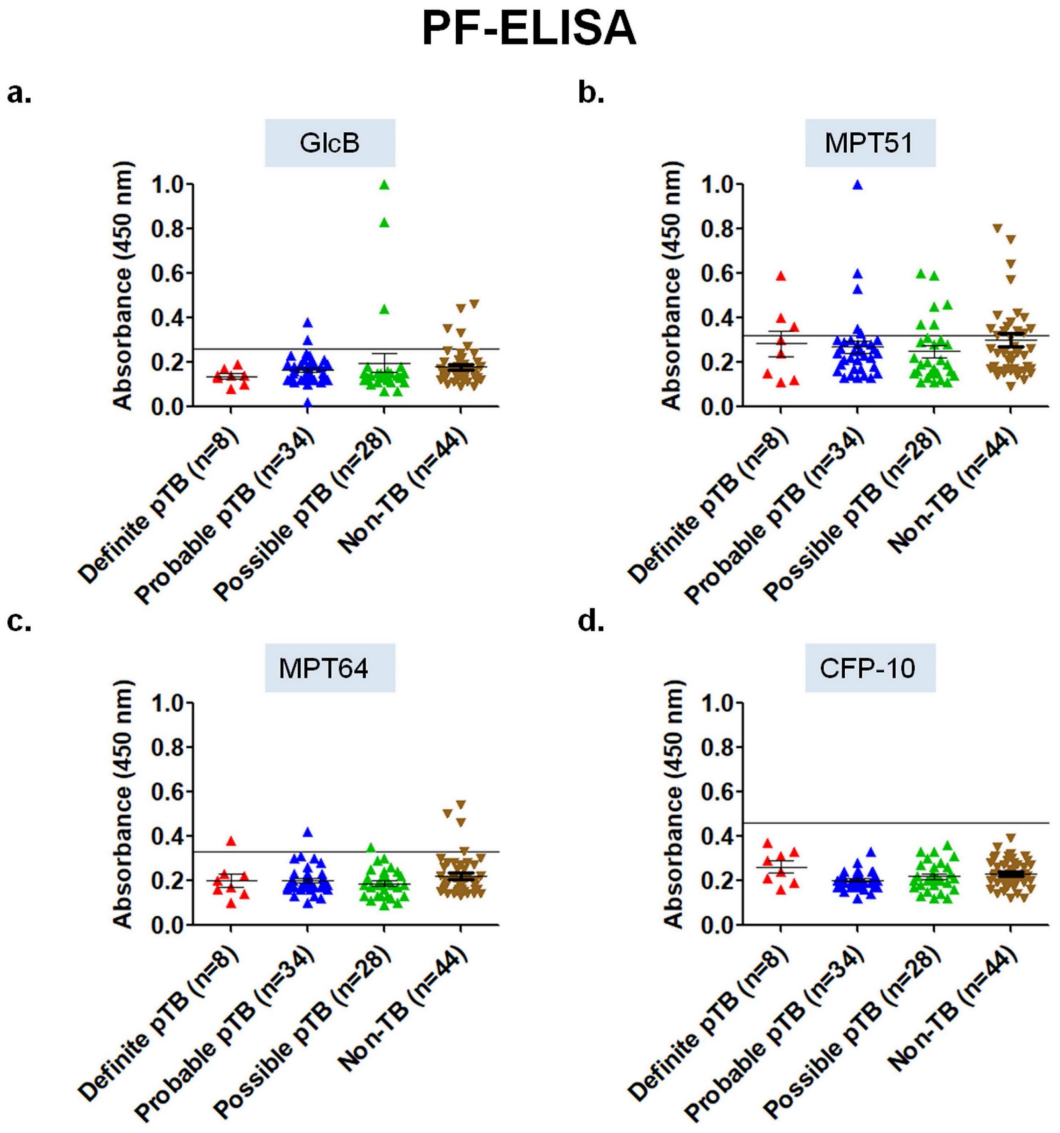
Scatterplots for OD_450_ values of ELISA in pleural fluid samples of 'Validation set' (n=114) of the study. a) GlcB, b) MPT51, c) MPT64, and d) CFP-10. Horizontal line across the graph indicates the cut-off calculated as mean+3SD of the OD_450_ values of Non-TB samples included in the 'Development set' (n=17) of the study.

**Performance of ELISA in pleural fluid samples for the 4 mycobacterial antigens.** Out of the 4 different antigens tested using conventional PF-ELISA, none of the antigens had an adequate diagnostic accuracy. In the 'Definite' pTB group, MPT64 and MPT51 antigen had a sensitivity of 12.5% (95% CI; 0.32-52.6) and 37.5% (95% CI; 8.5-75.5) with a specificity of 93.1% (95% CI; 81.3-98.5) and 63.6% (95% CI; 47.7-77.5) respectively. GlcB and CFP-10 PF-ELISA did not pick any sample in the 'Definite' pTB group. Overall, PF-ELISA had a poor performance in all the groups for pTB diagnosis (Table 2, Figure 3).

**Comparison of developed MNp-Ab-Ap assay with PF-ELISA and Xpert MTB/RIF assay in pleural fluid samples.** Of the two antigen-detection assays, MNp-Ab-Ap assay had a higher sensitivity and specificity as compared to conventional PF-ELISA. Xpert detected a total of n=6 samples which were included in the 'Definite' pTB group. The advantage of MNp-Ab-Ap assay over Xpert was observed in the 'Probable' and 'Possible' pTB groups wherein both MPT51 and MPT64-based assays detected 67.6% (n=23) samples in the 'Probable' pTB group and 46.4% (n=13) and 39.2% (n=11) samples respectively in the 'Possible' pTB group which were not detected by Xpert (Table 1).

**Figure 4:**
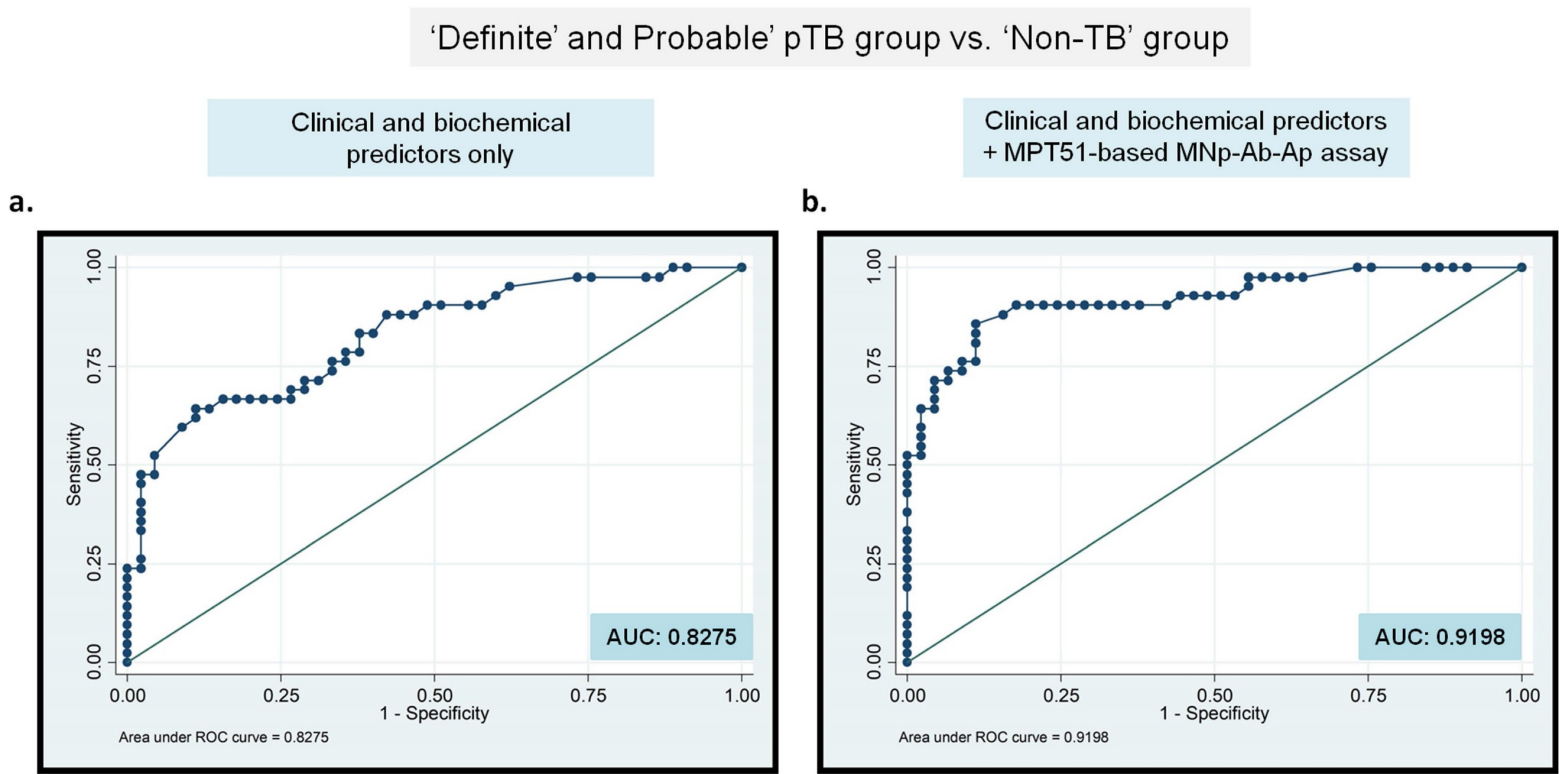
Binary logistic regression analysis for the 'clinical and biochemical' predictors along with MPT51-based MNp-Ab-Ap assay as a 'single test' in the 'Definite and Probable' pTB group. Receiver operating characteristic curves for MPT51-based MNp-Ab-Ap assay generated for 'Definite and Probable' pTB group vs. 'Non-TB' group. AUC: area under the curves.

**Binary Logistic Regression analysis demonstrates an additional benefit of MNp-Ab-Ap assay for pTB diagnosis.** Any new test that can help the clinician in establishing pTB diagnosis can improve case detection followed by improved treatment outcome. Therefore, the added benefit of the best-performing MPT51-based MNp-Ab-Ap assay ('new test') was assessed by generating a logistic regression model. ROC curves were generated first by modeling both, statistically significant clinical and cyto-biochemical predictors obtained in the study (fever, chest pain, loss of weight and appetite, cough, PF-sugar, PF-protein, PF-lymphocytic predominance and PF-ADA values; Supplementary Table S1 & S2) as a 'single test'. Next, ROC curves were again generated by including the 'new test' (MPT51-based MNp-Ab-Ap assay) along with the clinical and cyto-biochemical predictors in two ways, by including i) the combined results of 'Definite and Probable' pTB group (Figure 4) and ii) 'Total' pTB group (Supplementary Figure S2). The incremental impact of MPT51-based MNp-Ab-Ap assay was demonstrated by an increase in the area under the curve (AUC) from 0.8275 to 0.9198 in the combined 'Definite and Probable' pTB group (Figure 4) and from 0.9380 to 0.9964 in the 'Total' pTB group (Supplementary Figure S2).

## Discussion

There is no single accurate test that is currently available for pTB diagnosis 11. We, in this study assessed the diagnostic utility of antigen-detection in PF samples for pTB diagnosis by using 2 different approaches; a conventional indirect ELISA (PF-ELISA) and a novel MNp-Ab-Ap assay. Both assays were developed for the detection of 4 different mycobacterial antigens namely GlcB, MPT51, MPT64 and CFP-10 using PF samples. The results of conventional PF-ELISA were sub-optimal with a poor diagnostic accuracy (sensitivity; 0%-37.5%, specificity; 63.6%-100%) for all antigens tested in the study (Table 2). Previously published studies for antigen detection have also reported a variable sensitivity (8%-100%) and specificity (78%-100%) for pTB diagnosis using PF samples (Supplementary Table S3) 10, 11, 19-31. The inconsistent diagnostic performance of PF-ELISA might be attributed either to the presence of antigens in complexes, or in vesicles such as exosomes or antigens being diluted in large volumes of PF samples 18. Therefore, the MNp-Ab-Ap assay was developed aiming at capturing and concentrating the mycobacterial antigens present in PF samples. The features of MNp-Ab-Ap assay were; firstly, the use of antibody-conjugated MNPs to ensure efficient antigen-capture owing to their higher surface area-to-volume ratio, secondly, the magnetic property of the MNPs provided an advantage in simplifying the washing steps thereby eliminating the unwanted proteins of pleural fluid and thirdly, the addition of DNA aptamers as a 'detection reagent' for enhancing assay specificity. DNA aptamers have been reported to be excellent reagents in diagnosing various forms of EPTB (Supplementary Table S4) 11-13, 32-40. HspX-specific aptamers have successfully been used for pTB diagnosis using ALISA (sensitivity; 92.6% and specificity; 97.6%) 11, TBM diagnosis using electro-chemical sensing assay (sensitivity; 95% and specificity; 97.5%) 34 and ALISA (sensitivity; 100% and specificity; 91%) 13 and abdominal TB using ALISA (sensitivity; 84.2% and specificity; 96.3%) 12. GlcB-specific aptamers had a sensitivity and specificity of 50% and 98.1% by ALISA for abdominal TB diagnosis 12.

Out of 4 antigens tested by MNp-Ab-Ap assay, MPT51 proved to be the best antigen with 66.6% (95% CI; 50.4-80.4) sensitivity and 95.4% (95% CI; 85.1-99.4) specificity followed by MPT64, which had 64.2% (95% CI; 48-78.4) sensitivity and 86.3% specificity (95% CI; 72.6-94.8) in the combined 'Definite and Probable' pTB group (Table 1). Similar results were obtained in the 'Total pTB' group with a sensitivity of 58.5% (95% CI; 46.1-70.2) for MPT51 followed by MPT64 antigen [54.2% (95% CI; 41.9-66.2)] with similar specificity. The detection of both MPT51 and MPT64 antigens has been recently carried out by Dass et al in urine samples of patients with pleural effusion in our laboratory 18. If we compare the pTB groups in 2 studies, the sensitivity of MNp-Ab-Ap assay was considerably higher as compared to urine-based ELISA for both MPT51 and MPT64 antigens (MPT51: sensitivity; 66.6% vs. 19%, MPT64: 64.2% vs. 33.3%) in the combined 'Definite and Probable' pTB group. However, the specificity for MNp-Ab-Ap assay was higher for MPT51 but slightly lower for MPT64 than urine-based ELISA (Specificity; MPT51, 95.4% vs. 86.3%; MPT64, 86.3% vs 92.3%). The specificity of MPT51-based MNp-Ab-Ap assay (~95%) reached close to the minimum requirement of target product profile (TPP) of a newer diagnostic test for EPTB (specificity ≥ 98%) as described by the WHO. However, the assay had a sensitivity of ~ 60% to 67% which is although lower than the target set by TPP (target sensitivity; 80%), it is much higher than the reported sensitivity of existing WHO-endorsed tests for pTB 7, 8. Binary logistic regression analysis also indicated that the developed 'new test' could demarcate better between 'pTB' and 'Non-TB' cases and also provide an additional benefit over the currently used diagnostic algorithm for pTB (Figure 4 and Supplementary Figure S2).

This study had a number of strengths and limitations. One of the major strengths of the study was the development of a novel MNp-Ab-Ap assay which is an 'in-solution' tube-based assay that can be expanded to use larger sample volumes (~1 to 5 ml). The use of DNA aptamers as a 'detection reagent' led to an increased specificity of the assay by accurately binding with the target sites of the antigen. However, the application of this assay requires high precision for accurate results which could be overcome by adapting to automated systems for high throughput analysis. In addition, there have been several reports which have indicated the release of several small sized extracellular vesicles (EVs) in the body fluids which contain pathogen-specific proteins 18. Therefore, adding an additional step of lysis might release the antigens entrapped in the PF-EVs and could further lead to an increase in the sensitivity of the MNp-Ab-Ap assay.

We conclude that the MPT51-based MNp-Ab-Ap assay had a superior diagnostic accuracy over other antigens and has the potential to be translated in an automated system for pTB diagnosis. To the best of our knowledge, MNp-Ab-Ap assay is a novel approach that was developed and evaluated for the first time for pTB diagnosis. The challenge of attaining a perfect test for pTB is a mammoth task and although we tried to attain the target by using an innovative approach, there is still a need to find other novel host-specific or pathogen-specific biomarkers which could be evaluated for pTB diagnosis.

## Methods

**Ethical approval.** This study was approved from the Institutional Ethics Committee (IEC) of PGIMER, Chandigarh (NK/5266/PhD/786, dated 28/6/2019, PGI/IEC/2020/000563) and National Institute of Tuberculosis and Respiratory Diseases, New Delhi (NITRD/EC/2020/1541). PF samples were collected by the expert clinician only after obtaining the written informed consent from the patient or his/her guardian. The complete data in the study were reported referring to the STARD guidelines (Standards for Reporting of Diagnostic Accuracy Studies, Appendix A.1).

**Study participants.** This study included a total of n=153 study participants routinely visiting the out-patient department (OPD) of PGIMER, Chandigarh and NITRD, New Delhi with clinical presentations of fever, chest pain, breathlessness etc. and with pleural effusion. Pleural fluid (PF) samples were collected over a period of 2 years and seven months from May 2019 to December 2021. These samples were used for the development and evaluation of two assays; cell-free *Mycobacterium tuberculosis*-DNA based assay; cf*M.tb*-DNA based assay 9 and MNp-Ab-Ap assay (current study). The clinical, radiological and laboratory investigations were recorded in the case record form as approved by Ethical Committee (Appendix A.2) and maintained confidentially. Adult patients (> 14 years) were included and those who had a history of TB disease in preceding 2 years were excluded.

A total of n=153 study subjects were initially enrolled and n=131 were finally included in the study (Figure 1) and divided into 2 groups; a 'Development set' (n=17); used for assay development and 'Validation set' (n=114) used for evaluating the performance of the developed assays. The study participants were categorized into different categories on the basis of CRS as previously described [Supplementary Figure S3 9, 41, 42]. The 'Development set' (n=17) included 'Definite' pTB patients [n=2; Xpert MTB/RIF assay positive or AFB smear positive in sputum], 'Probable' pTB patients (n=7; CRS positive) and 'Non-TB' patients (n=8; malignancy). The 'Validation set' (n=114) included 'Definite' pTB patients (n=8) who were culture-positive for *M*.* tb*. or Xpert positive or smear positive in PF; 'Probable' pTB patients (n=34) who were clinically diagnosed as pTB and showed response to anti-tubercular therapy (ATT). In addition, a third category; 'Possible' pTB was also included (n=28), which had clinical and laboratory results indicative of pTB, however information for response to ATT was not available because the patient got lost to follow up and Non-TB patients (n=44) (Supplementary Figure S3). The data analysis was done in two ways by including PF samples i) of both 'Definite' pTB group and 'Probable' pTB group and ii) 'Definite' pTB, 'Probable' pTB and 'Possible' pTB; collectively called as 'Total' pTB group.

PF samples were routinely evaluated for cytology, biochemical analysis (glucose, protein and ADA levels), *M*.* tb*. culture and Xpert. The left-over PF sample was processed and stored as described recently [9, Figure 1]. *M*.* tb*. culture was performed using MGIT 960 culture system [Becton and Dickinson (BD), Franklin Lakes, New Jersey, United States] according to the manufacturer's instructions. Xpert MTB/RIF assay was carried out according to the manufacturer's instructions by adapting the previously reported protocol for PF 9.

**Protein purification and polyclonal antibody generation.** Recombinant expression vector pET23b vector encoding 'C' terminal (GlcB, MPT51 and CFP-10) or 'N' terminal (MPT64) hexa-histidine fusion of recombinant proteins of *M. tb*. were obtained from Biodefense and Emerging Infections Research Resources Repository (NIAID, NIH) and were available in the laboratory. *M. tb.* recombinant antigens; GlcB, MPT51, MPT64 and CFP-10 were overexpressed and purified from *E. coli* using Ni-NTA affinity chromatography as described previously 17, 18. Polyclonal antibodies for the antigens (anti-GlcB, anti-MPT51, anti-MPT64 and anti-CFP10) were raised in-house 43 using New Zealand White rabbits (male or female, < 10 weeks and 1.4-1.8 kg) only after obtaining ethical clearance from Institutional Animal Ethics Committee, PGIMER (100/IAEC/696). Briefly, a 2 ml pre-bleed was collected by piercing the marginal ear vein of the rabbit followed by immunizing the rabbit initially thrice with 100 μg of purified protein using Incomplete Freund's Adjuvant (IFA) (Sigma-Aldrich Corp.) at an interval of 7 days and bleed collection after 7 days of last immunization. Further, the booster dose of 70 μg was used for immunization every alternate week and 5-6 bleeds were collected post 7 days of each booster dose. IgG antibodies were purified using Melon^TM^ Gel IgG spin purification kit (Thermo Fisher Scientific Ltd.) and used for the development of conventional ELISA and magnetic nanoparticle antibody-conjugate-based aptamer-based assay (MNp-Ab-Ap assay) for all the 4 antigens.

**Preparation of magnetic nanoparticle antibody-conjugates.** The surface of the carboxy-modified magnetic nanoparticles namely Dynabeads MyOne^TM^ carboxylic acid (Invitrogen, Thermo Fisher Scientific Baltics UAB, Vilnius, Lithuania-02241) was conjugated with the purified polyclonal antibodies using 1-ethyl-3-(3-dimethylaminopropyl) carboiimide and N-hydroxysuccinimide (EDC-NHS) chemistry as per the manufacturer's instructions. Briefly, the magnetic nanoparticles were washed with 25 mM 4-morpholinoethanesulphonic acid (MES) buffer and activated by using reagents EDC and NHS in equal volumes. The EDC-NHS activated magnetic nanoparticles were used to immobilize the purified polyclonal antibodies (anti-GlcB, anti-MPT51, anti-MPT64 and anti-CFP-10 IgG) according to the binding capacity (50 μg ligand per ml) of the nanoparticles. The antibody-conjugated magnetic nanoparticles were used for the development of the MNp-Ab-Ap assay.

**Generation of aptamers.** The aptamers used in the study were prepared by Systemic Evolution of Ligand by Exponential Enrichment (SELEX) process as described previously 12. Briefly, 4 different ssDNA libraries including a central random sequence of 44 nucleotides flanked by fixed 18 nucleotide primer binding sequences were synthesized. Primers specific to the library were designed with a 5'-FAM/biotin label and 3'-ribo-adenine label. The ssDNA library and primers were obtained from Integrated DNA technologies, San Diego, USA (IDT, USA). Aptamers were selected by subtractive strategy on nitrocellulose membrane (NCM) with each round composed of a counter selection with control PF followed by purified protein. ssDNA oligonucleotides bound to purified protein were eluted by heating in nuclease free water and amplified by PCR as described previously 13. The amplified PCR products were precipitated overnight at -80 °C. The precipitated dsDNA was purified, treated with alkali [2.5 N NaOH (90 μl)] to cleave the rA-linkage in the reverse strand and converted to ssDNA and resolved on denaturing urea PAGE. The 5' FAM labelled forward band was excised and ssDNA was re-suspended in selection buffer (10 mM Tris-Cl, 10 mM MgCl₂, 50 mM KCl, 25 mM NaCl). SELEX was performed for 4 rounds and oligonucleotide pool obtained from each round was archived. The oligonucleotides binders of round 4 were amplified by PCR using primers without any label and ligated in pTZ57R/T vector by using InsTAclone PCR cloning kit (Thermo Fisher Scientific Ltd.) using manufacturer's instructions. Following transformation in *E. coli* strain DH5α, the resulting clones were sequenced and sequencing data was analyzed using BioEdit Sequence Alignment Editor Software (ver 7.2.5). Selected sequences were custom synthesized with 5' biotin labelled by IDT, USA. Any non-specific binding of generated aptamers was assessed by using purified protein (500 ng) and control pleural fluid by aptamer linked immobilized sorbent assay (ALISA).

MS_EPTB-10 aptamer for GlcB was previously developed by our group and its utility to detect pleural TB has been demonstrated (5'Biotin-TCGTCTCTTGCTGGCGGGTGCTTTGGTGGTAGGGGCGTCCTTGG) 11. However, the sequences of MPT51, MPT64 and CFP-10 aptamers are not being disclosed while their patents are pending.

**Figure 5:**
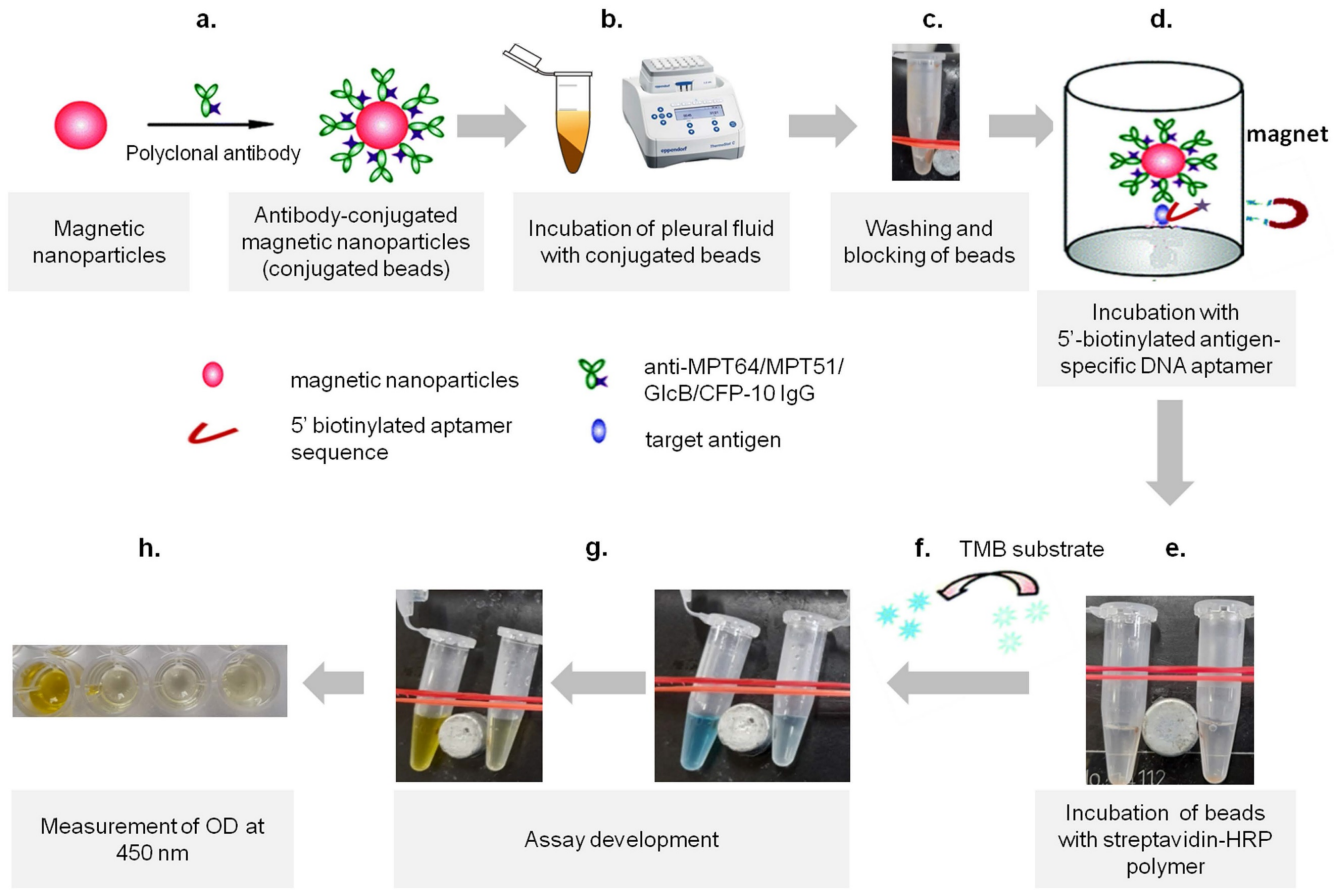
Development of MNp-Ab-Ap assay. The steps involved in the development includes a) the conjugation of polyclonal antibodies on the surface of the magnetic nanoparticles (conjugated beads), followed by b) incubation of appropriate dilution of pleural fluid with conjugated beads, c) washing of the beads by using an in-house magnetic apparatus and blocking of beads, d) incubation of beads with antigen-specific DNA aptamers, e) washing of beads and incubation with streptavidin-HRP polymer, f) development of assay by using 3,3',5,5'-tetramethylbenzidine (TMB) substrate, g) stopping the reaction using 50 μl of 2 N H_2_SO_4_ and h) absorbance measurement at 450 nm.

**Development of the magnetic nanoparticle antibody-conjugate aptamer-based assay (MNp-Ab-Ap assay).** The MNp-Ab-Ap assay was developed by using the antibody-conjugated magnetic nanoparticles which were first used to capture *M. tb*. antigens (GlcB, MPT51, MPT64 and CFP-10) in the PF samples. Next, the specific binding of 5' biotinylated aptamers with the MNp-Ab conjugates was assessed by using purified protein(s). Once this was successfully ascertained, the MNp-Ab-Ap assay was optimized for i) pleural fluid dilution to be used, ii) amount of antibody-conjugated MNPs to be used, iii) amount of aptamer to be used and iv) stringency of washing conditions. Although the limit of detection of the assays was not assessed, the final standardized conditions were evaluated on the 'Development set' (n=17) with 'pTB' (n=9) and 'Non-TB' cases (n=8) and then taken forward to be evaluated in the 'Validation set' in a blinded manner. Briefly, 2 μl (equivalent to 3.2 μg of conjugated anti-GlcB IgG, 2.8 μg of anti-MPT51 IgG & anti-MPT64 IgG and 1.2 μg anti-CFP10 IgG, as standardized) of antibody-conjugated Dynabeads™ were incubated with 200 μl of diluted PF sample (MPT64; 1:80 dilution and GlcB/MPT51/CFP-10; 1:60 dilution, as standardized) at 37 ˚C for 2 hours at 600 rpm in a shaking incubator followed by washing with selection buffer supplemented with 0.5% tween-20 to remove the unbound antigen and blocking with blocking buffer (5% bovine serum albumin supplemented with 0.25% tween-20 prepared in selection buffer). The resulting antibody-antigen complex was incubated with 10 pmol of 5' biotinylated aptamer for respective antigens followed by washing using selection buffer with 0.1% tween-20 and addition of streptavidin HRP polymer (1:4000) and development of the assay using 3, 3′, 5, 5′ -tetramethylbenzidine (TMB, BD Biosciences) substrate (Figure 5). Reaction was stopped by addition of 50 μl of 2 N H_2_SO_4_ and the absorbance was measured at 450 nm using multimode plate reader (TECAN, Zurich, Switzerland). Negative controls comprising of antigen control, aptamer control, and secondary antibody control were also included in each assay.

**Conventional ELISA.** ELISA was performed in a 96-well plate (Thermo Scientific Ltd. Nunc MaxiSorp™) by coating the PF samples diluted (1:40) in coating buffer (0.05 M sodium carbonate-bicarbonate buffer) and incubated at 4 ˚C overnight in a humidified chamber. The wells were blocked with 5% bovine serum albumin at 37 ^◦^C for 2 h followed by addition of diluted polyclonal antibody (anti-GlcB IgG 1:1500; anti-MPT51 IgG 1:1000; anti-MPT64 IgG 1:1500 and CFP-10 IgG; 1:1000). The wells were washed thrice with 1X PBS containing 0.1% tween-20 and once with 1X PBS followed by addition of 100 μl of donkey pAb to rabbit IgG-HRP; 1:12000 (Abcam) and incubated for 1 h at 37 ^◦^C. The wells were again washed followed by the addition of 100 μl of TMB substrate. Reaction was stopped by addition of 50 μl of 2 N H_2_SO_4_ and the absorbance was measured at 450 nm. Negative controls comprising of antigen control, primary antibody control, secondary antibody control and substrate control were also included in each assay.

**Statistical analysis.** The developed assays; MNp-Ab-Ap assay and conventional indirect ELISA were performed in the 'Validation set' (n=114 PF) in a blinded manner after coding the samples using a four-digit code. After the completion of all the assays, the codes were opened and the results were compiled and analyzed. The cut-off values for both the assays were calculated from mean+3SD of the OD_450_ values of the PF samples included in the 'Non-TB' group (n=8) of the 'Development set' (n=17). The cut-offs generated were applied to the 'Validation set' of the study and the performance of the assays were evaluated in the different groups of the study. The statistical significance of qualitative and quantitative features of the 'Definite and Probable' pTB group vs. 'Non-TB' group was calculated by Fisher's exact test and Mann Whitney test (p < 0.05 was considered as significant) respectively using GraphPad Prism version 5.0.0 for Windows, GraphPad Software, San Diego, California USA, www.graphpad.com”. The diagnostic accuracy for both the assays was calculated by web resources (https://www.medcalc.org/calc/diagnostic test.php). A binary logistic regression analysis was performed to determine whether the MNp-Ab-Ap assay was helpful in predicting pTB disease along with currently used diagnostic algorithm to better differentiate between 'pTB' and 'Non-TB' cases (Stata Statistical Software: Release 16. College Station, TX: StataCorp LLC, 2019). Odds ratios along with confidence interval were used as the measure of effect. A p-value of less than 0.05 was considered for significant association and the likelihood ratios along with Cox and Snell R square values were analyzed.

## Supplementary Material

Supplementary figures and tables, information.

## Figures and Tables

**Table 1:** Diagnostic performance of MNp-Ab-Ap assay for *M*.* tb*. antigens in pleural fluid samples.

Antigens	Patient Categories	TP^a^	FP^b^	FN^c^	TN^d^	Sensitivity (%) (95% CI)	Specificity (%) (95% CI)	PPV^e^ (%) (95% CI)	NPV^f^(%) (95% CI)	+LR^g^ (%) (95% CI)	-LR^h^ (%) (95% CI)	Accuracy (%) (95% CI)
GlcB	Definite pTB (n=8)	5	8	3	36	62.5 (24.4-91.4)	81.8 (67.2-91.8)	38.4 (21.5-58.7)	92.3 (82.9-96.7)	3.4 (1.5-7.8)	0.4 (0.1-1.1)	78.8 (65.3-88.9)
	Probable pTB (n=34)	11	8	23	36	32.3 (17.3-50.5)		57.8 (38.3-75.2)	61.0 (54.4-67.2)	1.7 (0.8-3.9)	0.8 (0.6-1.0)	60.2 (48.5-71.1)
	Possible pTB (n=28)	12	8	16	36	42.8 (24.4-62.8)		60 (41.2-76.2)	69.2 (61.3-76.1)	2.3 (1.1-5.0)	0.7 (0.4-0.9)	66.6 (54.5-77.3)
	Definite + Probable pTB (n=42)	16	8	26	36	38.1 (23.5-54.3)		66.6 (48.9-80.6)	58.0 (51.2-64.5)	2.1 (1-4.3)	0.7 (0.5-1.0)	60.4 (49.3-70.8)
	Total pTB (n=70)	28	8	42	36	40.0 (28.4-52.4)		77.7 (63.7-87.4)	46.1 (40.3-52)	2.2 (1.1-4.3)	0.73 (0.5-0.9)	56.1 (46.5-65.4)
MPT51	Definite pTB (n=8)	5	2	3	42	62.5 (24.4-91.4)	95.4 (85.1-99.4)	71.4 (36.7-91.4)	93.6 (85.6-97.2)	14.3 (3.3-61.7)	0.3 (0.16-0.96)	90.7 (79.7-96.9)
	Probable pTB (n=34)	23	2	11	42	67.6 (49.4-82.6)		92.0 (74.4-97.8)	80 (71.0-86.7)	15.5 (3.9-61.5)	0.3 (0.21-0.55)	83.7 (73.8-91.0)
	Possible pTB (n=28)	13	2	15	42	46.4 (27.5-66.1)		86.6 (61.2-96.3)	74.5 (67.3-80.6)	10.6 (2.6-43.8)	0.5 (0.3-0.8)	77.0 (65.7-86.0)
	Definite + Probable pTB (n=42)	28	2	14	42	66.6 (50.4-80.4)		93.3 (78-98.2)	75.8 (67.1-82.8)	15.3 (3.8-60.4)	0.3 (0.2-0.5)	81.8 (72.1-89.2)
	Total pTB (n=70)	41	2	29	42	58.5 (46.1-70.2)		95.3 (83.9-98.7)	60.2 (53.2-66.8)	13.4 (3.4-53)	0.4 (0.3-0.5)	73.2 (64.2-81.0)
MPT64	Definite pTB (n=8)	4	6	4	38	50 (15.7-84.3)	86.3 (72.6-94.8)	40 (19.4-64.8)	90.4 (82.4-95)	3.6 (1.3-10.1)	0.5 (0.2-1.1)	80.7 (67.4-90.3)
	Probable pTB (n=34)	23	6	11	38	67.6 (49.4-82.6)		79.3 (63.7-89.3)	77.5 (67.6-85)	4.9 (2.2-10.8)	0.3 (0.2-0.6)	78.2 (67.4-86.7)
	Possible pTB (n=28)	11	6	17	38	39.2 (21.5-59.4)		64.7 (43.3-81.4)	69 (61.8-75.4)	2.8 (1.2-6.9)	0.7 (0.5-0.9)	68.0 (56.0-78.5)
	Definite + Probable pTB (n=42)	27	6	15	38	64.2 (48-78.4)		81.8 (67.4-90.7)	71.7 (62.4-79.4)	4.7 (2.1-10.2)	0.4 (0.2-0.6)	75.5 (65.1-84.2)
	Total pTB (n=70)	38	6	32	38	54.2 (41.9-66.2)		86.3 (74.4-93.2)	54.2 (47.2-61.1)	3.9 (1.8-8.6)	0.5 (0.4-0.7)	66.6 (57.1-75.2)
CFP10	Definite pTB (n=8)	2	5	6	39	25 (3.1-65)	88.6 (75.4-96.2)	28.5 (8.5-63.1)	86.6 (81.1-90.7)	2.2 (0.5-9.44)	0.8 (0.5-1.2)	78.8 (65.3-88.9)
	Probable pTB (n=34)	5	5	29	39	14.7 (4.9-31.0)		50 (23.9-76)	57.3 (53.0-61.5)	1.2 (0.4-4.1)	0.9 (0.8-1.1)	56.4 (44.7-67.6)
	Possible pTB (n=28)	11	5	17	39	39.2 (21.5-59.4)		68.7 (46-84.9)	69.4 (62.5-75.8)	3.4 (1.3-8.8)	0.6 (0.5-0.9)	69.4 (57.4-79.7)
	Definite + Probable pTB (n=42)	7	5	35	39	16.6 (6.9-31.3)		58.3 (32.5-80.2)	52.7 (48.4-56.9)	1.4 (0.5-4.2)	0.9 (0.7-1.1)	53.4 (42.4-64.3)
	Total pTB (n=70)	18	5	52	39	25.7 (16-37.5)		78.2 (59-90)	42.8 (38.6-47.1)	2.2 (0.9-5.6)	0.8 (0.7-1.0)	50 (40.4-59.5)

^a^TP: true positives, ^b^FP: false positives, ^c^FN: false negatives, ^d^TN: True negatives, ^e^PPV: positive predictive value, ^f^NPV: negative predictive value, ^g^+LR: positive likelihood ratio, ^h^-LR: negative likelihood ratio

**Table 2:** Diagnostic performance of developed conventional ELISA for 4 mycobacterial antigens in pleural fluid samples.

Antigens	Patient Categories	TP^a^	FP^b^	FN^c^	TN^d^	Sensitivity (%) (95% CI)	Specificity (%) (95% CI)	PPV^e^ (%) (95% CI)	NPV^f^(%) (95% CI)	+LR^g^ (%) (95% CI)	-LR^h^ (%) (95% CI)	Accuracy (%) (95% CI)
GlcB	Definite pTB (n=8)	0	5	8	39	0 (0-36.9)	88.6 (75.4-96.2)	0	82.9 (81.4-84.4)	0	1.1 (1.0-1.2)	75 (61-85.9)
	Probable pTB (n=34)	2	5	32	39	5.8 (0.7-19.6)		28.5 (7.6-65.9)	54.9 (51.5-58.2)	0.5 (0.1-2.5)	1.0 (0.9-1.2)	52.5 (40.9-63.9)
	Possible pTB (n=28)	3	5	25	39	10.7 (2.2-28.2)		37.5 (13.4-69.8)	60.9 (56.9-64.8)	0.9 (0.2-3.6)	1.0 (0.8-1.1)	58.3 (46.1-69.8)
	Definite + Probable pTB (n=42)	2	5	40	39	4.7 (0.5-16.1)		28.5 (7.5-66.1)	49.3 (46.2-52.5)	0.4 (0.09-2)	1.0 (0.9-1.2)	47.6 (36.7-58.7)
	Total pTB (n=70)	5	5	65	39	7.1 (2.3-15.8)		50 (23.4-76.5)	37.5 (34.6-40.4)	0.6 (0.2-2)	1.0 (0.9-1.2)	38.6 (29.6-48.1)
MPT51	Definite pTB (n=8)	3	16	5	28	37.5 (8.5-75.5)	63.6 (47.7-77.5)	15.7 (6.6-33.2)	89.8 (75.7-90.9)	1.0 (0.3-2.7)	0.9 (0.5-1.7)	59.6 (45.1-72.9)
	Probable pTB (n=34)	9	16	25	28	26.4 (12.8-44.3)		36 (22.1-52.6)	52.8 (45.3-60.2)	0.7 (0.3-1.4)	1.1 (0.8-1.5)	47.4 (36.0-59)
	Possible pTB (n=28)	6	16	22	28	21.4 (8.3-40.9)		27.2 (14.3-45.7)	56 (48.6-63.1)	0.5 (0.2-1.3)	1.2 (0.9-1.6)	47.2 (35.3-59.3)
	Definite + Probable pTB (n=42)	12	16	30	28	28.5 (15.7-44.5)		42.8 (28.8-58.1)	48.2 (41-55.6)	0.7 (0.4-1.4)	1.1 (0.8-1.5)	46.5 (35.6-57.5)
	Total pTB (n=70)	18	16	52	28	25.7 (16.0-37.5)		52.9 (39.1-66.2)	35 (29.2-41.1)	0.7 (0.4-1.2)	1.1 (0.9-1.5)	40.3 (31.2-49.9)
MPT64	Definite pTB (n=8)	1	3	7	41	12.5 (0.32-52.6)	93.1 (81.3-98.5)	25 (3.8-73.8)	85.4 (81.6-88.5)	1.8 (0.2-15.4)	0.9 (0.7-1.2)	80.7 (67.4-90.3)
	Probable pTB (n=34)	1	3	33	41	2.9 (0.07-15.3)		25 (3.5-75.4)	55.4 (52.9-57.8)	0.4 (0.05-3.9)	1.0 (0.9-1.15)	53.8 (42.1-65.2)
	Possible pTB (n=28)	0	3	28	41	0 (0.0-12.3)		-	59.4 (57.4-61.3)	0	1.0 (0.9-1.1)	56.9 (44.7-68.5)
	Definite + Probable pTB (n=42)	2	3	40	41	4.7 (0.5-16.1)		40.0 (10.4-79.1)	50.6 (48.0-53.2)	0.7 (0.1-3.9)	1.0 (0.9-1.1)	50 (39.0-60.9)
	Total pTB (n=70)	2	3	68	41	2.8 (0.3-9.9)		40 (10.3-79.3)	37.6 (35.5-39.4)	0.4 (0.07-2.4)	1.0 (0.9-1.1)	37.7 (28.8-47.2)
CFP-10	Definite pTB (n=8)	0	0	8	44	0 (0-36.9)	100 (91.9-100)	-	84.6 (84.6-84.6)	1.0 (1.0-1.0)	-	84.6 (71.9-93.1)
	Probable pTB (n=34)	0	0	34	44	0 (0-10.2)		-	56.4 (56.4-56.4)	1.0 (1.0-1.0)	-	56.4 (44.7-67.6)
	Possible pTB (n=28)	0	0	28	44	0 (0-12.3)		-	61.1 (61.1-61.1)	1.0 (1.0-1.0)	-	61.1 (48.8-72.3)
	Definite pTB+ Probable pTB (n=42)	0	0	42	44	0 (0-8.4)		-	51.1 (51.1-51.1)	1.0 (1.0-1.0)	-	51.1 (40.1-62.1)
	Total pTB (n=70)	0	0	70	44	0 (0-5.1)		-	38.6 (38.6-38.6)	1.0 (1.0-1.0)	-	38.6 (29.6-48.1)

^a^TP: true positives, ^b^FP: false positives, ^c^FN: false negatives, ^d^TN: True negatives, ^e^PPV: positive predictive value, ^f^NPV: negative predictive value, ^g^+LR: positive likelihood ratio, ^h^-LR: negative likelihood ratio
