# Supplementary material for: Development and assessment of a novel magnetic nanoparticle antibody-conjugate and aptamer-based assay (MNp-Ab-Ap assay) for the rapid diagnosis of pleural tuberculosis
Source: Nanotheranostics. 2025 Jan 1;9(1):20–30. doi: 10.7150/ntno.95332 (PMC11667566; doi:10.7150/ntno.95332)
Supplement: Supplementary file 1 — Supplementary figures and tables, information. [file ntnov09p0020s1.pdf]

Supplementary information

Table S1. Characteristics features and clinical symptoms as presented by the study participants[#]

Qualitative characteristics	'Definite pTB' (n=8) n (%)	'Probable pTB' (n=34) n (%)	'Possible pTB' (n=28) n (%)	'Non-TB' (n=44) n (%)	p-value*
**Male /Female (M%/F%)**	8/0 (100/0)	14/20 (41.1/58.8)	18/10 (64.2/35.7)	28/16 (63.6/36.3)	**-**
**Fever**	4 (50)	22 (64.7)	11(39.2)	10 (22.7)	**0.0009**
**Weight loss**	3 (37.5)	13 (38.2)	5 (17.8)	7 (15.9)	**0.0482**
**Chest pain**	3 (37.5)	14 (41.1)	8 (28.5)	8 (18.1)	**0.0185**
**Loss of appetite**	4 (50)	18 (52.9)	7 (25)	8 (18.1)	**0.0028**
**Night sweats**	1 (12.5)	9 (26.4)	1 (3.5)	0	**0.0004**
**Cough**	2 (25)	19 (55.8)	15 (53.5)	12 (27.2)	**0.0269**
**Shortening of breath**	2 (25)	9 (26.4)	16 (57.1)	18 (40.9)	0.8154
**Hypertension**	1 (12.5)	1 (2.9)	2 (7.1)	6 (13.6)	0.2663
**Diabetes mellitus**	1 (12.5)	1 (2.9)	3 (10.7)	6 (13.6)	0.4845
**Smoking**	1 (12.5)	2 (5.8)	5 (17.8)	3 (6.8)	1.000

# Values represents the number of patients showing clinical symptoms in each category; values in brackets indicate the percentage of patients presenting the clinical symptom.

* Statistical significance was calculated between 'Definite and Probable' pTB group vs. Non-TB group and $p < 0.05$ was considered as statistically significant and has been represented in bold.

Table S2. Laboratory findings of the study participants in different categories#

Laboratory Investigations	'Definite' pTB' (n=8)	'Probable' pTB' (n=34)	'Possible' pTB' (n=28)	'Non-TB' (n=44)	p-value*
**Hematological parameters**					
**Hemoglobin (g/dL)**	12.4 (11.2-13.4)	11.9 (10-12.8)	10.5 (8.3-12.3)	9.5 (8.4-11.8)	**0.0213**
**TLC (x 10^9/L)**	9.9 (9.4-12.6)	8.8 (7.3-12.7)	9.7 (7.2-13.7)	10.9 (7.2-16.6)	0.5639
**Neutrophils**	75 (73.1-82.1)	71 (62.2-75.8)	81 (72-86.3)	84.3 (73.6-89.4)	**0.0111**
**Leukocytes**	12.3 (8.4-16.1)	24 (16.5-27)	11 (6.9-17.1)	7.9 (4.6-13.4)	**0.0036**
**Monocytes**	2.6 (2.0-3.2)	7.7 (5-8.7)	5.6 (3.6-8.9)	6.8 (4.6-8.5)	0.6805
**Eosinophils**	4.2 (4.0-4.4)	1.7 (1.0-3.2)	1.1 (0.6-2.4)	0.7 (0.35-2.2)	0.2502
**Basophils**	0.5 (0.4-0.6)	0.4 (0.3-0.4)	0.2 (0.1-0.7)	0.4 (0.2-0.5)	0.4207
**Conjugated Bilirubin (mg/dL)**	0.3 (0.2-0.3)	0.24 (0.18-0.32)	0.26 (0.12-0.44)	0.29 (0.2-0.6)	0.4072
**Total Bilirubin (mg/dL)**	0.5 (0.4-0.5)	0.5 (0.3-0.6)	0.53 (0.4-0.8)	0.5 (0.38-0.95)	0.3812
**AST (U/L)**	28 (25-29.8)	33.2 (27.2-42.4)	45.6 (27.5-77.1)	27 (17.3-43.8)	0.1757
**ALT (U/L)**	24 (18-26.9)	23.3 (17.7-41.2)	38.9 (16.1-66.2)	17.7 (10.7-61.3)	0.2434
**ALP (U/L)**	135.9 (118.8-152.9)	112 (103-150.6)	149 (116-205)	113 (91-144)	0.8827
**Urea (mg/dL)**	18.1 (14.8-20)	24.1 (20.5-40.9)	39 (23.1-111)	42 (30.9-107)	**0.0003**
**Creatinine (mg/dL)**	0.6 (0.5-0.7)	0.7 (0.5-1.1)	0.86 (0.57-3.8)	0.9 (0.6-5.4)	**0.0165**
**Uric acid (mg/dL)**	4.3 (4.2-4.4)	6.1 (4.9-7.6)	5.1 (3.1-9.2)	3.5 (2.4-5.4)	**0.0589**
**Blood sugar (mg/dL)**	94.9 (94.8-95)	110.5 (91-142.5)	117 (99.5-134.5)	120 (99.5-174.5)	0.4639
**Serum protein**	6 (5.8-6.2)	7.6 (6.8-7.9)	6.3 (5.4-6.9)	6.4 (5.8-6.9)	**0.0007**
**Cyto-biochemical analysis**					
**Cytology (cells/ml)**	1139 (584.5-1693.5)	1741(328-3200)	800 (430-2300)	713.5 (271.2-1311)	0.2163
**Lymphocytes (%)**	NA	96 (70-100)	63.1 (40-86.9)	59.1 (36.5-84)	**0.0025**
**PF sugar (mg/dL)**	3 (2.5-6.7)	82 (65-116.9)	94 (48-119.5)	112.3 (96.2-128.6)	**0.0037**
**PF protein (g/dL)**	4.5 (2.9-6.0)	5.1 (4.3-5.6)	4.5 (3.7-4.8)	3.5 (2.5-4.7)	**0.0007**
**PF/ serum protein**	0.06 (0.03-0.1)	0.6 (0.3-0.6)	0.6 (0.5-0.7)	0.4 (0.03-0.67)	0.9903
**ADA (U/L)**	90.2 (74.0-137.2)	53.1 (39.2-61.5)	25 (12-34)	10 (7-20)	**<0.0001**

Values represents the median values of the laboratory findings in each group; the values in the brackets represent the 1st and 3rd quartile range (IQI;IQIII).

*statistical significance was calculated between 'Definite and Probable' pTB group vs. 'Non-TB' group using Mann Whitney's test. *p*-value < 0.05 was considered as statistically significant and has been represented in bold. NA- data not available. TLC- total leucocyte count, AST- Aspartate amino transferase, ALT- Alanine amino transferase, ALP- Alkaline phosphatase, ADA- adenosine deaminase assay.

Table S3. Studies assessing the antigen detection assays for pleural TB diagnosis in literature

Author	Year	Ref no.	Country	Sample type[#]	Antigen[*]	No. of samples	Method used[$]	Sensitivity	Specificity
Yan et al	2023	23	China	PF	LAM	210	Sandwich ELISA	30.6	94
Mustafa et al	2020	28	Norway	PF	Secreted antigen or LAM	41	Immuno-cytochemistry	56	78
Kumari et al	2019	11	India	PF	HspX	98	ELISA	23.6	97.7
Liang et al	2019	22	China	PF	LAM	155	anti-LAM antibody assay	35.5	96.9
Xiaoxin et al	2017	24	China	PF	ESAT-6, CFP-10	34	ELISA	73.5, 67.6	
Tedele et al	2014	29	Ethiopia	PF	MPT64	63	Immuno-staining	81.0	88.2
Liu et al	2012	25	China	PF	MPT64	82	ELISA	79.5	97.7
Feng et al	2011	26	China	PF	ESAT-6, CFP-10	71	ELISA	86.8, 76.3	100, 83.3
Kalra et al	2009	27	India	EPTB (4 PF)	ESAT6, CFP-10, MPT64	35	ELISA	31	95
Kamaldeen et al	2008	30	South Africa	Pleural biopsy	MPT-64	25	Immuno-staining	81	100
Tiwari et al	2007	31	India	PF	Glycolipid antigen	9	TB/M card	100	100
Dheda et al	2009	21	SA	PF	LAM	12	ELISA	8	100
Anie et al	2007	19	India	PF	Glycoprotein	69	ELISA	100	100
Banchuin et al	1990	20	Thailand	PF	PPD	26	ELISA	12	100

[#]PF: pleural fluid, EPTB: extrapulmonary TB. [*]LAM: lipoarabinomannan, ESAT: early secretory antigenic target, CFP: culture filtrate protein, PPD: purified protein derivative. [$]ELISA: enzyme-linked immunosorbent assay.

Table S4. Studies employing aptamers as a diagnostic reagent for diagnosis of different forms of tuberculosis.

Author	Year	Ref no.	Method#	Antigens*	Sample$	Disease^	Sensitivity %	Specificity %
Bethu et al	2023	32	ALISA	HspX	Sputum, GA, CSF, PF, PCF	PTB , EPTB	62.9	73.7
				MPT64			58	76.3
Kumari et al	2022	12	ALISA	HspX	AF	Abdominal TB	84.2	96.3
				GlcB			50	98.1
Zhou et al	2021	33	IHC	ManLAM	FFPE tissues	PTB, EPTB	86.3	92.8
Das et al	2019	34	ECS	HspX	CSF	TBM	95	97.5
Sypabekova et al	2019	36	EIS-aptasensor	MPT64	sputum	PTB	76.4	100
					serum		88.2	100
Lavania et al	2018	37	ALISA	HspX	sputum	PTB	94.1	100
			ECS				92.3	91.2
Kumari et al	2019	11	ALISA	HspX	PF	Pleural TB	92.6	97.6
Dhiman et al	2018	13	ALISA	HspX	CSF	TBM	100	91
Sypabekova et al	2017	38	ELONA	MPT64	sputum	PTB	91.3	90
Tang et al	2016	39	ELONA	ManLAM	sputum	PTB	92.7	98.7
					Serum		83	95.3
					serum	EPTB	88.7	94.4
Tang et al	2014	35	ELONA	ESAT-6	Serum	PTB	100	94
				CFP-10		EPTB	89.6	94.1
Zhu et al	2012	40	Aptamer ELISA	MPT64	Serum	PTB	64.4	99.4

#ALISA: aptamer-linked immunosorbant assay, IHC: immunohistochemistry, ECS: electrochemical sensor, ELONA: enzyme-linked oligonucleotide assay. *ManLAM: mannos-capped lipoarabinomannan. $ GA: gastric aspirate, CSF: cerebrospinal fluid, PF: pleural fluid, PCF: pericardial fluid, FFPE: formalin-fixed paraffin embedded tissues, ^PTB: Pulmonary TB, EPTB: Extrapulmonary TB, TBM: tuberculous meningitis

Supplementary Figures

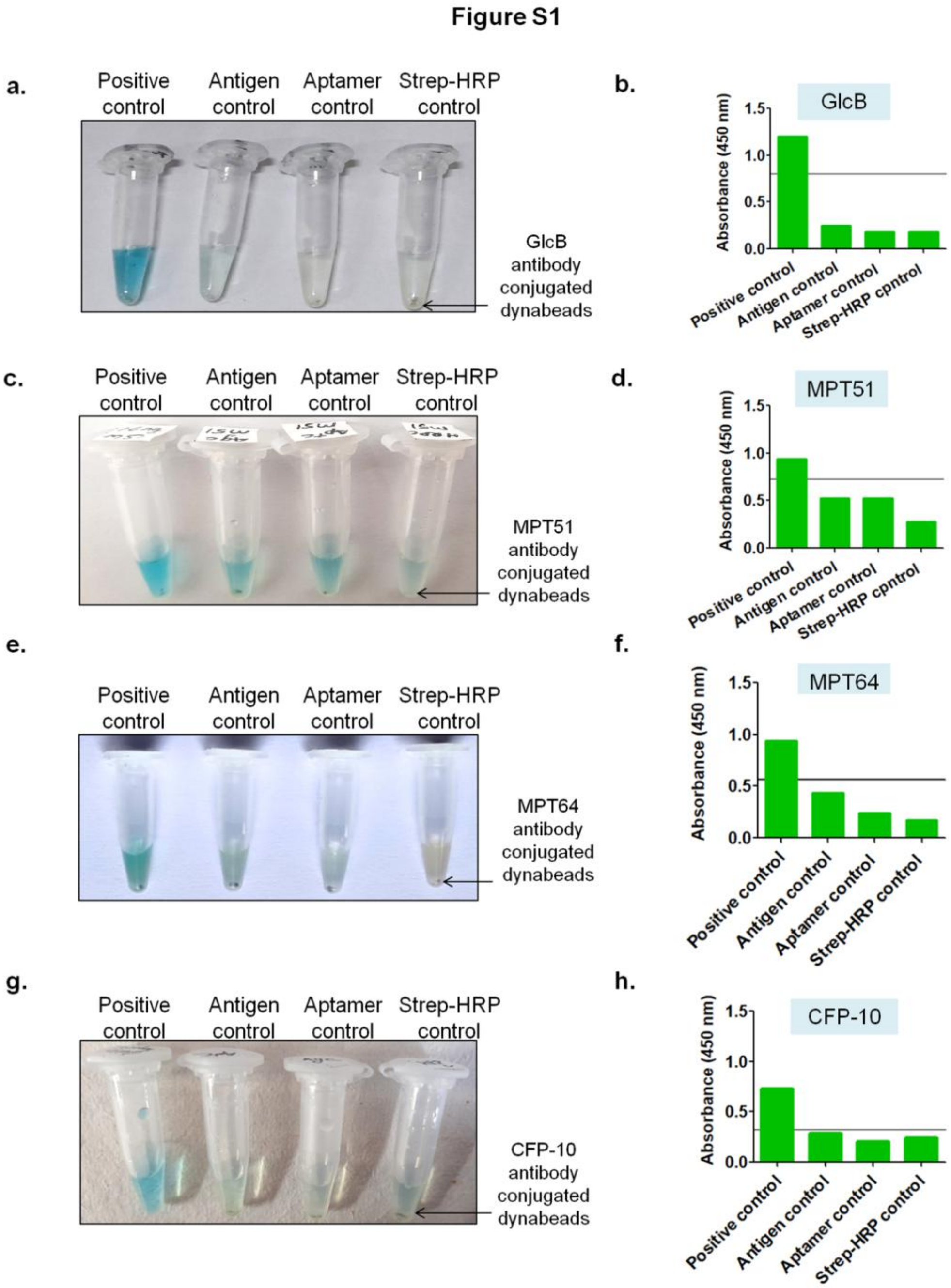

Figure S1. Assessment of the specific binding of 5’ biotinylated aptamers with the MNp-Ab conjugates using purified protein(s). a & b) GlcB, c & d) MPT51, e & f) MPT64 and g & h) CFP-10. Black horizontal line indicates the mean+3SD of the negative controls i.e antigen control, aptamer control and streptavidin HRP control used in the assay. The positive control included purified protein GlcB (16 ng), MPT51 (16 ng), MPT64 (2 ng) and CFP-10 (64 ng) used in the assay.

Figure S2

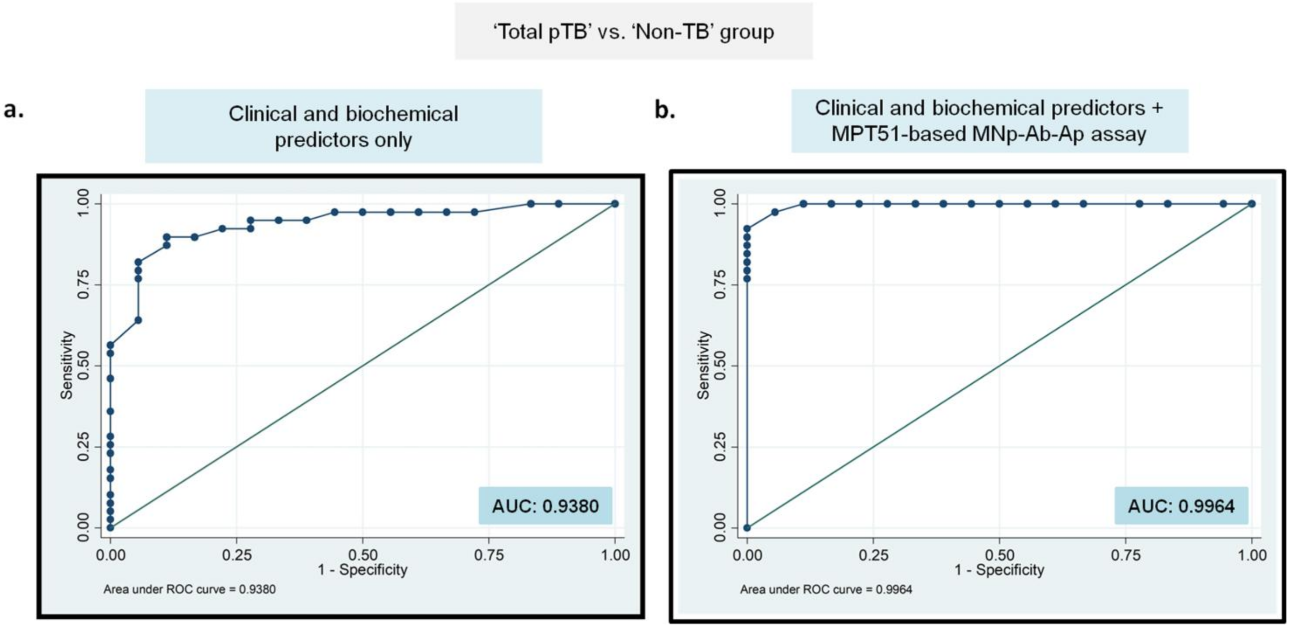

Figure S2. Binary logistic regression analysis for the 'clinical and biochemical predictors along with MPT51-based MNp-Ab-Ap assay as a 'single test' in the 'Total pTB' group. Receiver operating characteristic curves for MPT51-based MNp-Ab-Ap assay generated for 'Total' pTB group ('Definite', 'Probable' and Possible' pTB group) vs. 'Non-TB' group. AUC: area under the curves.

Figure S3

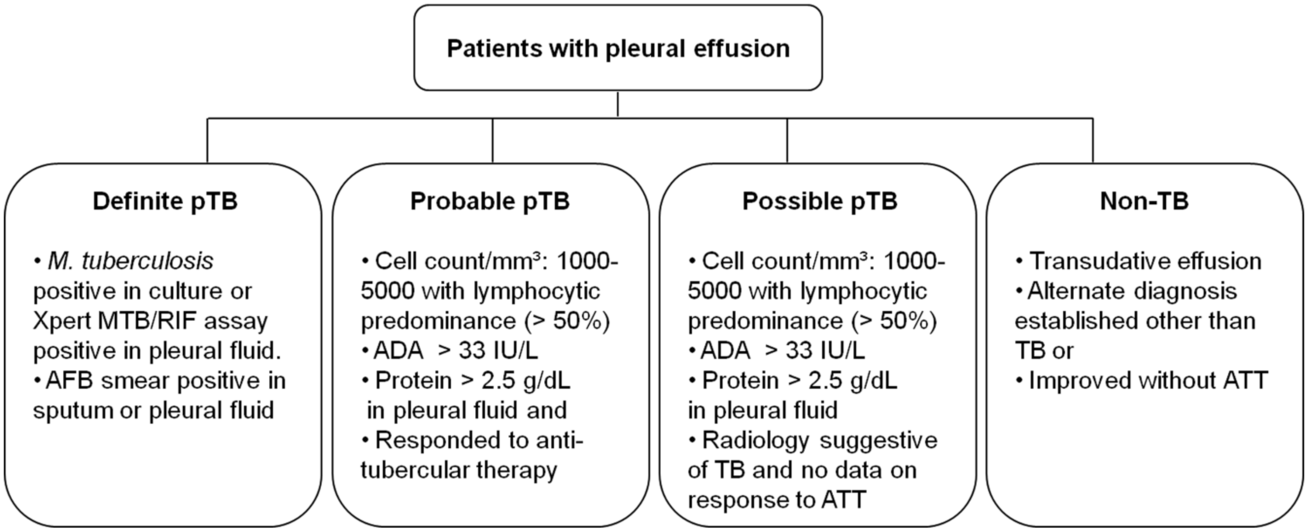

Figure S3. Composite Reference Standard (CRS) used for the categorization of the study participants

Appendix A.1: STARD checklist

Section and Topic	No	Item	Reported on page #
**TITLE OR ABSTRACT**	**1**	Identification as a study of diagnostic accuracy using at least one measure of accuracy (such as sensitivity, specificity, predictive values, or AUC)	Page # 1, 2 and 3<br>The title and abstract identify the manuscript as a study of diagnostic accuracy.
**ABSTRACT**	**2**	Structured summary of study design, methods, results, and conclusions (for specific guidance, see STARD for Abstracts)	Page # 2 and 3<br>An abstract including details about objective, methods, results and conclusions.
	**3**	Scientific and clinical background, including the intended use and clinical role of the index test	Page # 4 and 5<br>The Introduction focuses on the manuscript in a wider context. It includes a brief review of the key references and the need for the development of 'Magnetic nanoparticle antibody conjugate and aptamer-based assay (MNp-Ab-Ap assay)'.
	**4**	Study objectives and hypothesis	Page # 5<br>This study focuses on the development and evaluation of a novel MNp-Ab-Ap assay for the detection of 4 different *M. tb* specific antigens (GlcB, MPT51, MPT64 and CFP-10) in PF samples wherein antibody-conjugated magnetic nanoparticles (MNPs) were used to efficiently capture the antigens followed by the detection of resulting antigen-antibody complexes by antigen-specific DNA aptamers and comparison of

			the developed assay with conventional indirect ELISA. This study hypothesized that *M. tb* antigens present in PF samples could be used as a biomarker for pleural TB diagnosis and their efficient capture can lead to an improved sensitivity of the antigen-detection assays.
Study design	**5**	Whether data collection was planned before the index test and reference standard were performed (prospective study) or after (retrospective study)	Page # 11 and 12<br>Data collection was planned before the index test and reference standard were performed. This was a prospective study performed in a blinded manner.
Participants	**6**	Eligibility criteria	Page # 11 and 12<br>Adult patients (> 14 years) with radiological evidence of pleural effusion were included in the study.
	**7**	On what basis potentially eligible participants were identified (such as symptoms, results from previous tests, inclusion in registry)	Page # 11, 12 and Table S1<br>Adult patients (> 14 years) with radiological evidence of pleural effusion were included in the study. Symptoms are described in Table S1.
	**8**	Where and when potentially eligible participants were identified (setting, location and dates)	Page # 11<br>Pleural fluid (PF) was collected from the out-patient department (OPD) of PGIMER, Chandigarh and National Institute of Tuberculosis and Respiratory Diseases (NITRD), New Delhi with clinical presentations of fever, chest pain, breathlessness etc. and with pleural effusion. Pleural fluid (PF) samples were collected over a period of 2 years and seven months from May 2019 to December 2021 after obtaining ethical clearance from the Institutional Ethics Committee. We obtained written

			informed consent from participants in accordance with ethical guidelines from participating institutions.
	**9**	Whether participants formed a consecutive, random or convenience series	Page # 11<br>Patients presenting with complaints of chest pain, fever, breathlessness etc. with radiological evidence of pleural effusion were included consecutively in the study.
**Test Methods**	**10a**	Index test, in sufficient detail to allow replication	Page # 12, 13, 14, 15 and 16<br>We have given details of the protocols in above mentioned page numbers which can be sufficiently replicated while using the protocols.
	**10b**	Reference standard, in sufficient detail to allow replication	Page # 11, 12, Supplementary Figure S3<br>Composite reference standard (CRS) was used as a reference standard for pleural TB diagnosis.
	**11**	Rationale for choosing the reference standard (if alternatives exist)	Since there are no universal guidelines that exist for categorization of pleural TB patients and culture-based test has a poor sensitivity of pleural TB diagnosis, we used composite reference standard (CRS) formulated for this study.
	**12a**	Definition of and rationale for test positivity cut-offs or result categories of the index test, distinguishing pre-specified from exploratory	Page # 17<br>Receiver operating characteristics (ROC) curves were plotted and cut-off values were determined by using the results of samples included in the 'Development set' (n=17) and generated cut-offs were applied to the 'Validation set' (n=114) of the study for evaluation of the developed assays. Diagnostic accuracy was calculated using GraphPad Prism version 5.0 for Windows (GraphPad

			Software, US).
	**12b**	Definition of and rationale for test positivity cut-offs or result categories of the reference standard, distinguishing pre-specified from exploratory	NA
	**13a**	Whether clinical information and reference standard results were available to the performers/ readers of the index test	Page # 17 and Table S1, Table S2<br>The clinical information and reference standard results were not available to the performers/readers of the index test as the study was carried out in a blinded manner.
	**13b**	Whether clinical information and index test results were available to the assessors of the reference standard	Page # 17 and Table S1, Table S2<br>The clinical information and index test results were not available to the assessors of the reference standard as the study was carried out in a blinded manner.
**Analysis**	**14**	Methods for estimating or comparing measures of diagnostic accuracy	Page # 17<br>Included in statistical analysis.
	**15**	How indeterminate index test or reference standard results were handled	Fig. 1<br>Samples with indeterminate results were excluded from the study.
	**16**	How missing data on the index test and reference standard were handled	Fig. 1<br>Samples with indeterminate/missing results were excluded from the study.
	**17**	Any analyses of variability in diagnostic accuracy, distinguishing pre-specified from exploratory	Page # 17<br>included in statistical analysis
	**18**	Intended sample size and how it was determined	NA

**RESULTS**			
Participants	**19**	Flow of participants, using a diagram	Fig. 1
	**20**	Baseline demographic and clinical characteristics of participants	Supplementary information Page # 1 and 2 (Table S1 & Table S2)
	**21a**	Distribution of severity of disease in those with the target condition	NA
	**21b**	Distribution of alternative diagnoses in those without the target condition	NA
	**22**	Time interval and any clinical interventions between index test and reference standard	NA
Test results	**23**	Cross tabulation of the index test results (or their distribution) by the results of the reference standard	Supplementary information Page # 1 and 2 (Table S1 & Table S2).
	**24**	Estimates of diagnostic accuracy and their precision (such as 95% confidence intervals)	Page # 21, 22, 23 and 24 (Table 1 and 2, Figure 2 and 3).
	**25**	Any adverse events from performing the index test or the reference standard	NA
**DISCUSSION**			
	**26**	Study limitations, including sources of potential bias, statistical uncertainty, and generalisability	Page # 10 Included in the 'Strengths and limitations of study' under discussion section.
	**27**	Implications for practice, including the intended use and clinical role of the index test	Page # 9 and 10 Test details are included in discussion section.

**OTHER INFORMATION**			
	**28**	Registration number and name of registry	NA
	**29**	Where the full study protocol can be accessed	NA
	**30**	Sources of funding and other support; role of funders	Details are given in ‘Funding information’ section.

A.2 DATA COLLECTION SHEET

S.No.

Name : **OPD card Date:** **Age :** **Sex :**

CR No: **Date of sample withdrawn:** **Address:**

Phone No.

Final Diagnosis- ………………………………………………………………………………………

Clinical details- **Lab. Investigations and Radiology-**

**History**	**1 if yes, 0 if No, Duration (days)**	**Hemat. Exam.**	
Fever		Haemoglobin	
Weight loss		TLC	
Chest pain		DLC	
Loss of appetite		ESR	
Night sweats		Platelet	
Cough		**LFT (Total)**	
Expectoration		D. Bil	
Hemoptysis		I. Bil	
HTN		AST (SGOT)	
DM		ALT (SGPT)	
Past h/o TB		ALP	
Family h/o TB		**KFT-** Urea	
Alcohol		Creatinine	
Smoking		Uric acid	
Shortening of breath		Sodium	
Night sweats		Potassium	
HIV status		Concomitant blood sugar (mg/dL)	
**Any other**			
**Examination**	**1 if yes, 0 if No**	Total protein	
Lymphadenopathy		Albumin	
Chest exam		A:G ratio	
CVS exam		Mantoux	
Hepatomegaly		CXR	
Splenomegaly			
Any other finding		CECT	

Sample Volume		

PLEURAL FLUID ANALYSIS

Fluid analysis (fill in relevant columns for each fluid type)	Pleural fluid	Provisional diagnosis-
Color		
Cytology : total cells/ml		
Cytology : differential		
Malignant Cytology		Final Diagnosis-
Sugar		
Protein		
ADA		Treatment
Albumin		
Light's criteria		
LDH		ATT (Y/N)

MICROBIOLOGICAL INVESTIGATIONS-

Culture (Pyogenic)		
Sensitivity		
Smear Microscopy		
TB Culture		
Xpert MTB/Rif assay		

Follow up:

ATT treatment: Yes/No, (If yes: then Duration ……...days); Weight Gain..................(yes / no)

Date of Start of ATT: ………………

Date of Completion of ATT:……………

…………………………….
Signature of Senior Resident
Name:……………………..

Any other test done to confirm follow up:
